# Community-associated methicillin-resistant *Staphylococcus aureus* infections in Aboriginal children attending hospital emergency departments in a regional area of New South Wales, Australia: a seven-year descriptive study

**DOI:** 10.5365/wpsar.2017.8.1.014

**Published:** 2017-12-12

**Authors:** Susan Thomas, Kristy Crooks, Fakhrul Islam, Peter D Massey

**Affiliations:** a University of Newcastle, Newcastle, NSW, Australia.; b Hunter New England Local Health District, Population Health, Wallsend, NSW, Australia.; c College of Medicine and Dentistry, James Cook University, Cairns, Queensland, Australia.

## Abstract

**Objective:**

Community-associated methicillin-resistant *Staphylococcus aureus* (CA-MRSA) can cause bacterial skin infections that are common problems for Aboriginal children in New South Wales (NSW). MRSA is not notifiable in NSW and surveillance data describing incidence and prevalence are not routinely collected. The study aims to describe the epidemiology of CA-MRSA in Aboriginal children in the Hunter New England Local Health District (HNELHD).

**Methods:**

We linked data from Pathology North Laboratory Management System (AUSLAB) and the HNELHD patient administration system from 33 hospital emergency departments. Data from 2008–2014 for CA-MRSA isolates were extracted. Demographic characteristics included age, gender, Aboriginality, rurality and seasonality.

**Results:**

Of the 1222 individuals in this study, 408 (33.4%) were Aboriginal people. Aboriginal people were younger with 45.8% aged less than 10 years compared to 25.9% of non-Aboriginal people. Most isolates came from Aboriginal people who attended the regional Tamworth Hospital (193/511 isolates from 149 people). A larger proportion of Aboriginal people, compared to non-Aboriginal people, resided in outer regional (64.9% vs 37.2%) or remote/very remote areas (2.5% vs 0.5%). Most infections occurred in summer and early autumn. For Aboriginal patients, there was a downward trend through autumn, continuing through winter and spring.

**Discussion:**

Aboriginal people at HNELHD emergency departments appear to represent a greater proportion of people with skin infections with CA-MRSA than non-Aboriginal people. CA-MRSA is not notifiable in NSW; however, pathology and hospital data are available and can provide valuable indicative data to health districts for planning and policy development.

## Introduction

Community-associated methicillin-resistant *Staphylococcus aureus* (CA-MRSA) can cause bacterial skin infections that are common health problems for many Australian Aboriginal and Torres Strait Islander (hereafter Aboriginal) children and families in rural areas in New South Wales (NSW). (*1*) The term CA-MRSA distinguishes the infection from MRSA acquired through health-care settings including hospitals.

Typical infections caused by CA-MRSA include skin and soft tissue infections, boils, impetigo, cellulitis and larger abscesses. CA-MRSA is contagious, transmitted by skin-to-skin contact from infected lesions, contact with contaminated objects or close contact with asymptomatic carriers. (*2*) Groups at higher risk of infection include children and young adults, Aboriginal people and people of lower socioeconomic status. (*2*) Indigenous populations in Canada, the United States of America and in Pacific island nations have also been associated with a high risk of infection with CA-MRSA attributed possibly to social and financial disadvantage. (*3*) Associated risk factors include crowded living conditions with poor housing infrastructure and lack of access to facilities for adequate personal cleansing, pre-existing skin conditions and previous antimicrobial drug treatment. (*4*) MRSA is not notifiable in NSW and hence, surveillance data describing its incidence and prevalence are not routinely collected. Such data would be invaluable in the planning, implementation and evaluation of public health programmes designed to prevent and control CA-MRSA.

Early diagnosis and treatment for CA-MRSA is recommended as delays may lead to serious complications including septicaemia. (*4*) Recommended treatment includes incision and drainage of wounds, cautious use of antibiotics (when indicated by pathology and/or when lesions are larger than 5 cm, with systemic sepsis or patients who are immunocompromised), personal cleansing measures (covering draining wounds, regular showering and handwashing and not sharing personal items such as linen, towels, razors), consideration of staphylococcal skin load reduction with bleach baths or formal decolonization for those with recurrent boils and/or household involvement and maintaining close follow-up by primary health care (PHC) services. (*5*) These guidelines may not adequately take into account important sociocultural factors or ways of living in Aboriginal communities where CA-MRSA infections can impact health, quality of life and contribute to poor school attendance. (*1*)

We used routinely collected pathology data from wound and/or skin swabs collected in emergency departments of hospitals in the Hunter New England Local Health District (HNELHD) to describe the epidemiology of CA-MRSA in Aboriginal children and young people. For the purpose of this study, we defined CA-MRSA as not ‘hospital onset’ or ‘health-care-associated community origin’. This definition was taken from the study from where our data was collected. (*6*) It reflects both the Centers for Disease Control and Prevention (CDC) classification (*7*) and accommodates Australian practices and limitations of the data set (**Table 1**). Results will inform the development of health policy and community-based programmes to reduce the incidence and prevalence of the infection.

**Table 1:** Comparison of study definitions of hospital origin (HO) and health-care-associated community onset (HACO) methicillin-resistant *Staphylococcal aureus* (MRSA) with the Centers for Disease Control and Prevention (CDC) definitions

MRSA category	Study definition	CDC definition8	Rationale for difference
HO MRSA	Culture obtained within 48 hours following admission or 48 hours following discharge	Culture obtained within 96 hours of admission	Study definition reflects Australian definition of hospital-associated infection as used in National Health Performance Authority reports
HACO MRSA	Not HO MRSA, and culture was obtained within 365 days of a previous hospital admission or within 365 days of receiving dialysis	Includes patients with a central vascular catheter (CVC). Includes residence in a long-term care facility.	Unable to determine presence of CVC from collected data. In line with Australian conventions, long-term care facilities (residential aged-care facilities) should not be classified as health-care facilities.

## Methods

**Fig. 1:**
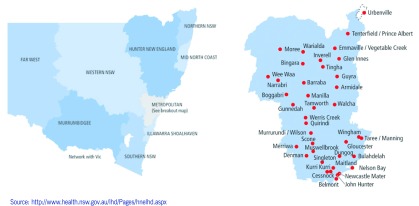
Map of Hunter New England Local Health District and hospitals, NSW, 2017

The study setting was HNELHD, which covers a large regional area of northern NSW. The region is largely rural with one metropolitan centre in Newcastle (**Fig. 1**). (*8*) In 2011, the total population of HNELHD was 875 546 including 46 955 Aboriginal people (5.4%). Of the total Aboriginal population, almost half were aged under 20 years (23 207 or 49.4%) compared to just a quarter of the non-Aboriginal population (203 575 or 24.6%). (*9*)

We used routinely collected administrative data linked by laboratory number from a previous study examining the changing epidemiology of *Staphylococcus aureus* in HNELHD. This data set included pathology data from the Pathology North Laboratory Management System (AUSLAB) and patient characteristics and hospitalization data from the HNELHD patient administration system. The study period was 1 January 2008 to 31 December 2014. Five of the district’s 38 hospitals were excluded as they did not use AUSLAB or data were not available for comparison.

From the complete data set of 81 133 positive *S. aureus* isolates, all those classified as CA-MRSA (*n* = 7789) were identified. Isolates from residents of residential aged-care facilities (*n* = 398), those not from skin or wound swabs (*n* = 768), those for which Aboriginal and/or Torres Strait Islander status was not recorded (*n* = 79) and those aged 20 years or older (*n* = 4335) were removed. Swabs collected within two days of an emergency department presentation were included. Those taken outside of the two-day period were considered to be from presentations to general practitioners (GP) and were excluded (*n* = 658). This left 1551 isolates for CA-MRSA from 1222 individuals.

The number of isolates by hospital emergency department was used as a measure of burden of disease. From the 1222 individuals with a first isolate of CA-MRSA (as opposed to isolates of repeated testing), demographic characteristics including age, gender and Aboriginality were described. Street addresses were used to assign a Statistical Area Level 2 (SA2) location to each individual and to classify rurality. Seasonality of infection was described using date of first isolate.

Proportions and counts were analysed using Stata 14^®^ and Excel 2010^®^. The Australian Statistical Geography Standard (ASGC) Remoteness Structure 2011 was used to classify metropolitan, regional and remote/very remote settings. The ASGC: Volume 1, 2011 population counts were used to calculate rates within SA2 areas. (*10*) Geocoding was conducted using the Geocoder Optimised for Population Health Epidemiology and Research from NSW Ministry of Health.

## Results

Of the 1222 individuals in this study, 408 (33.4%) were Aboriginal people and 814 (66.6%) were non-Aboriginal people (**Table 2**). Overall, Aboriginal people were younger with 45.8% aged less than 10 years compared to 25.9% of non-Aboriginal people. There was a higher proportion of males than females (ratio 1.3/1.0).

**Table 2:** Number and proportion of individuals aged under 20 years with hospital emergency department wound/skin swabs with CA-MRSA, by age and Aboriginality, Hunter New England Local Health District, 2008–2014

Age (years)	Aboriginal		non-Aboriginal	
	n	%	n	%
0–4	87	21.3	91	11.2
5–9	100	24.5	120	14.7
10–14	108	26.5	209	25.7
15–19	113	27.7	394	48.4
**Total**	**408**	**100.0**	**814**	**100.0**

Of the 1551 isolates, 511 (32.9%) were from Aboriginal people. The highest number of isolates from Aboriginal people came from those who attended the regional hospital in Tamworth (193/511 isolates from 149 people) and John Hunter Hospital in Newcastle (69/511 isolates from 55 people). For non-Aboriginal people, most isolates came from people who attended John Hunter Hospital (256/1040 isolates from 196 people) and Maitland Hospital (176/1040 isolates from 139 people), which lies within close proximity to Newcastle (data not shown).

The proportion of Aboriginal people residing in outer regional and remote areas (64.9% and 2.5%, respectively) was higher compared to that of non-Aboriginal people (37.2% and 0.5%, respectively) (**Table 3**).

**Table 3:** Number and proportion of individuals aged under 20 years with hospital emergency department wound/skin swabs with CA-MRSA by region, Hunter New England Local Health District, 2008–2014*

Region	Aboriginal		non-Aboriginal	
	n	%	n	%
Major city	78	19.3	249	30.9
Inner regional	54	13.3	254	31.5
Outer regional	263	64.9	300	37.2
Remote/very remote	10	2.5	4	0.5
**Total**	**405**	**100.0**	**807**	**100.0**

* Australian Standard Geographical Classification (ASGC)

Some missing data and individuals residing outside HNELHD account for totals less than full data set in Table 2.

Aboriginal people resided largely in the SA2 locations of the regional centre of Tamworth (*n* = 146, 36.0%) and in Armidale (*n* = 34, 8.4%) (**Table 4**).

**Table 4:** Number and proportion of individuals aged under 20 years with hospital emergency department wound/skin swabs with CA-MRSA by top 12 geographic locations, Hunter New England Local Health District, 2008–2014

Location (SA2)	Aboriginal		non-Aboriginal	
	*n*	%	*n*	%
**Tamworth – West**	**66**	**16.3**	**25**	**3.1**
Tamworth – East	40	9.9	46	5.7
Armidale	34	8.4	22	2.7
Tamworth – North	25	6.2	20	2.5
Maitland – East	16	4.0	31	3.8
Tamworth Region	15	3.7	39	4.8
Raymond Terrace	11	2.7	47	5.8
Muswellbrook	11	2.7	10	1.2
Kurri Kurri – Abermain	10	2.5	27	3.3
Inverell	9	2.2	12	1.5
Tenterfield	9	2.2	4	0.5
Cessnock	9	2.2	25	3.1
**Total top 12 sites**	**255**	**63**	**308**	**38**

**Fig. 2:**
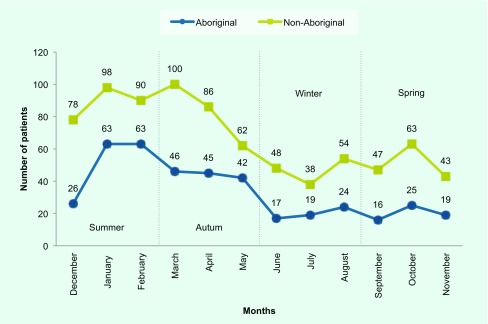
Individuals aged under 20 years with hospital emergency department wound/skin swabs with CA-MRSA, by season, Hunter New England Local Health District, 2008–2014

Seasonal analysis of CA-MRSA isolates in the study period showed most cases occurred in summer and early autumn. For Aboriginal patients, there was a downward trend through autumn, continuing through winter and spring. Apart from a peak in early autumn for non-Aboriginal patients, the trend was similar for both groups (**Fig. 2**).

### Ethics

Ethics approval was obtained for this study by HNELHD Human Research Ethics Committee (12/12/12/5.08).

## Discussion

Overall, Aboriginal people accounted for 33.4% of CA-MRSA first isolates in the study period while accounting for just 10.2% of the total population aged under 20 years in the wider HNELHD population. (*9*) Aboriginal people in this study tended to live in outer regional, rural or remote areas, especially in the north-western of the state of NSW including Tamworth, Armidale and Inverell. The emergency department at the regional centre of Tamworth had the highest number of CA-MRSA isolates identified in Aboriginal people. An important and less described finding was that most isolates were from summer months and early autumn when it is warmer, more humid and when children are more likely to be playing outdoors. A similar trend was reported for paediatric patients in Rhode Island, USA, where approximately 1.85 times as many CA-MRSA infections per emergency department visit occurred in summer and autumn. (*11*)

Published data describing the local epidemiology of CA-MRSA are limited, impeding informed policy development and health service planning that can address the burden of skin infection experienced by Aboriginal families. This study provides new information that can be used to direct health resources to areas within HNELHD where needs are higher and when the number of CA-MRSA infections are high, such as summer and early autumn. There are advantages in using readily available administrative data as a source of surveillance information to inform practice. Data are readily available for timely analysis, collected routinely and are easily linked for analysis of trends and patient characteristics. These methods can be reproduced by other local health districts that have access to linked pathology and hospital data. In this way, more information about CA-MRSA infections in NSW can be uncovered.

It is known that Aboriginal people are more likely to attend hospital emergency departments if access to community-based, culturally safe and appropriate PHC is limited. This may occur more often in rural and remote areas. (*12*) It is important to ensure that staff working in emergency departments provide a culturally safe place for Aboriginal people to seek care about skin infections. Currently the NSW treatment guidelines for skin infections may not incorporate the consideration of important, associated and interwoven contributors such as social, economic, housing and environmental factors in the management of infections. (*1*) Tailoring treatment guidelines to respond to these social determinants of health for Aboriginal people in rural areas, who access PHC in acute settings, may be an effective step to reducing recurrence of disease. (*1*) The emergency department could be an important setting for improving skin health through sharing of health information, initiating referrals and arranging for follow-up of children with skin diseases.

CA-MRSA surveillance models, coordinating both hospital and community activities at the local and state level have been proposed as a way of providing a more comprehensive epidemiological assessment. (*13*) Local hospitals, general practitioners and other health facilities would be required to collect data from patients and contacts while state authorities would aggregate and disseminate surveillance reports. The resources required to implement such a system would be significant (personnel, materials, time, storage and transportation) and underreporting may be another limitation. (*13*) A five-year incidence study of CA-MRSA in remote communities in Canada found high rates of infection with 25% of infections being re-infection. The study concluded that surveillance was important in understanding antibiotic resistance and the changing profile of CA-MRSA. (*14*) While this debate continues, the surveillance of CA-MRSA in NSW could be improved by adopting a uniform surveillance definition for community association. Surveillance alone will not solve the problem of bacterial skin infections caused by CA-MRSA. Health and other services need to address the contextual factors which cause persistent infection, especially the social determinants, (*15*) normalization of the problem of bacterial skin infections, transgenerational trauma and access to culturally safe and appropriate PHC. (*1*)

This study has some limitations. Using passive surveillance through administrative hospital data has not captured all cases. Many people with CA-MRSA use PHC, including Aboriginal Community Controlled Health Services, community health centres and GPs, from which data were not available for inclusion. Data from some hospitals were not included as they did not use AUSLAB or data were not available due to changes in information systems. These limitations imply that the number of people experiencing CA-MRSA is higher than reported here. As our numerators were uncertain, we were unable to either calculate population rates or use statistical methods to compare results between groups or locations that would have provided additional useful data.

CA-MRSA is not notifiable in NSW; however, pathology and hospital administration data can be linked to assist in the estimation of the magnitude and scope of the problem. Implementing routine surveillance requires further consideration in light of the costs and limitations of notification of CA-MRSA. Timely dissemination of these data can assist in service planning, policy development and evaluation. Targeted prevention activities can be designed in collaboration with Aboriginal health services for rural hospital emergency departments before and during peak seasons. This would be particularly valuable in rural and remote areas where, in the absence of adequate, culturally safe PHC, many Aboriginal people utilize hospital emergency departments for management of bacterial skin infections caused by CA-MRSA. Further research using administrative pathology data can be undertaken to better understand the phenotypes and antibiotic sensitivity of CA-MRSA affecting Aboriginal children in NSW. Due to changing patterns of antibiotic resistance, genotyping of regular samples from hospital and community settings would be valuable.
